# A laboratory-based beam tracking x-ray imaging method achieving two-dimensional phase sensitivity and isotropic resolution with unidirectional undersampling

**DOI:** 10.1038/s41598-023-35901-2

**Published:** 2023-05-29

**Authors:** G. Lioliou, C. Navarrete-León, A. Astolfo, S. Savvidis, D. Bate, M. Endrizzi, C. K. Hagen, A. Olivo

**Affiliations:** 1 Department of Medical Physics and Biomedical Engineering, University College London, Malet Place, London, WC1E 6BT UK; 2 Nikon X-Tek Systems Ltd, Tring, HP23 4JX Herts UK

**Keywords:** Biomedical engineering, X-rays, Imaging techniques

## Abstract

Beam tracking X-ray Phase Contrast Imaging is a “Shack-Hartmann” type approach which uses a pre-sample mask to split the x-rays into “beamlets” which are interrogated by a detector with sufficient resolution. The ultimate spatial resolution is determined by the size of the mask apertures, however achieving this resolution level requires “stepping” the sample or the mask in increments equal to the aperture size (“dithering”). If an array of circular apertures is used (which also provides two-dimensional phase sensitivity) instead of long parallel slits, this stepping needs to be carried out in two directions, which lengthens scan times significantly. We present a mask design obtained by offsetting rows of circular apertures, allowing for two-dimensional sensitivity and isotropic resolution while requiring sample or mask stepping in one direction only. We present images of custom-built phantoms and biological specimens, demonstrating that quantitative phase retrieval and near aperture-limited spatial resolutions are obtained in two orthogonal directions.

## Introduction

Contrast in conventional x-ray images depends on the attenuation of x-rays passing through matter; conventional x-ray computed tomography (CT) provides information on the internal structure of materials in three-dimensions based on the attenuation signal^1^. Both planar x-rays (radiography) and CT are routinely used in a variety of applications, including medicine and material science. However, they suffer from low contrast in cases where the sample is weakly attenuating (e.g., biological tissue) and/or consists of multiple materials with similar attenuation.

Overcoming the limitations of attenuation-based x-ray imaging has been the subject of extensive research during the past decades. One approach is to utilize, in image formation, the phase shift that x-rays experience while passing through matter, which gives rise to refraction effects (the refraction angle is proportional to the first derivative of the phase shift^2,3^). X-ray phase contrast imaging (XPCI) and tomography (XPC-CT) are powerful techniques that have many advantages over attenuation-based methods; in particular, they enable significantly higher contrast^4^. This results in an increase in contrast-to-noise (CNR) ratio for the same detected x-ray statistics, ultimately allowing details that are invisible to conventional x-ray imaging to be detected, and different materials to be more easily discriminated. In addition, phase-based contrast can be maintained at high x-ray energies, which reduces the amount of dose deposited in the sample^3,5^, an advantage particularly useful in biomedical imaging.

Imaging techniques that exploit x-ray phase in image formation include propagation-based imaging methods^6^, analyzer-based imaging methods^7^, speckle-based imaging methods^8^, crystal-based interferometric methods^9^, grating-based interferometric methods^10^, and grating-based non-interferometric methods^11^. These methods use different experimental setups to generate phase sensitivity, and consequently their requirements very in terms of x-ray beam spatial and temporal coherence. Some attempts to quantitatively compare different XPCI methods have been made in the past^12–14^.

The subject of this paper is a grating-based non-interferometric imaging method. This category of methods employs modulators, typically masks with alternating absorbing and transmitting septa, upstream of the sample, which structure the x-ray beam into an array of beamlets with negligible mutual overlap. The sample’s attenuation and refraction then lead to an intensity reduction and to a lateral shift of the beamlets, respectively. Sensitivity to the latter is achieved by using either a second mask at the detector (edge illumination^11^), or a detector with a sufficiently small pixel size to individually resolve the beamlets (beam tracking^15^). Although the requirement for small pixel size detectors limits its field-of-view, beam tracking has the significant advantage that attenuation and refraction signals are retrieved from a single frame. It should be noted here that, both sensing mechanisms, edge illumination and beam tracking, enable also the retrieval of the dark field (small-angle scattering) signal; however, this was considered beyond the scope of this work, which focuses on the unidirectional scanning allowed by a new mask design. We initially tested beam tracking XPCI with synchrotron radiation^16^, then translated it to a laboratory setup^15^; in both cases, one-dimensional phase sensitivity was achieved using a mask with long, parallel slits. This technique was further developed for CT^17,18^, for two-dimensional phase sensitivity using a mask with round apertures^19,20^, and by combining both of these advances with synchrotron radiation^21^ and in a compact laboratory set-up^22^. It should be noted that direct resolution of an array of beamlets with a detector with sufficient resolution shares similarities with the Shack-Hartman wavefront sensor (which, however, uses lenses), and indeed other groups developed similar concepts, even earlier on^23,24^.

A common feature of mask-based methods is that the parts of the sample covered by the mask septa do not contribute to the image, which is however also what provides the option for aperture-limited resolution^25^. This higher resolution can be accessed through a “dithering” scheme, consisting of scanning the sample (or the mask) in steps equal to the aperture size, acquiring images at all steps, and recombining them. Dithering is required only along the horizontal direction when a mask with parallel slits is used (1D structured beam). In this case, the phase sensitivity in the direction parallel to the slits is lacking. A significantly more extensive sample stepping is required for a 2D structured beam (enabling two-dimensional phase sensitivity), as the sample (or the mask) needs to be stepped both horizontally and vertically; a non-isotropic resolution is otherwise achieved. The fully dithering scheme has been investigated in 2D beam-tracking CT with both synchrotron radiation^21^ and a laboratory setup^22^ and has been proven effective in improving the spatial resolution to values equal to the aperture size (= dithering step size) in both directions. In both these cases, dithering steps in a 2D grid were acquired; since in CT the dithering process must be applied at each angle, this increases both the acquisition time and the complexity of the acquisition procedure.

Here, we propose a mask design for beam tracking that benefits from two-dimensional phase sensitivity and isotropic spatial resolution, without the need for extensive scanning in a 2D grid. In the following, we first describe the implementation of the approach, then present planar images and CT scans of both custom-built samples and of a complex biological one (a rat heart). Although not fully exploited in this first proof-of-concept study, the method allows fully illuminating a sample with a 2D array of circular apertures while using unidirectional scanning; this concept is expanded on in the Supplementary Materials.

## Results and discussion

### 2D beam tracking and unidirectional dithering

**Figure 1:**
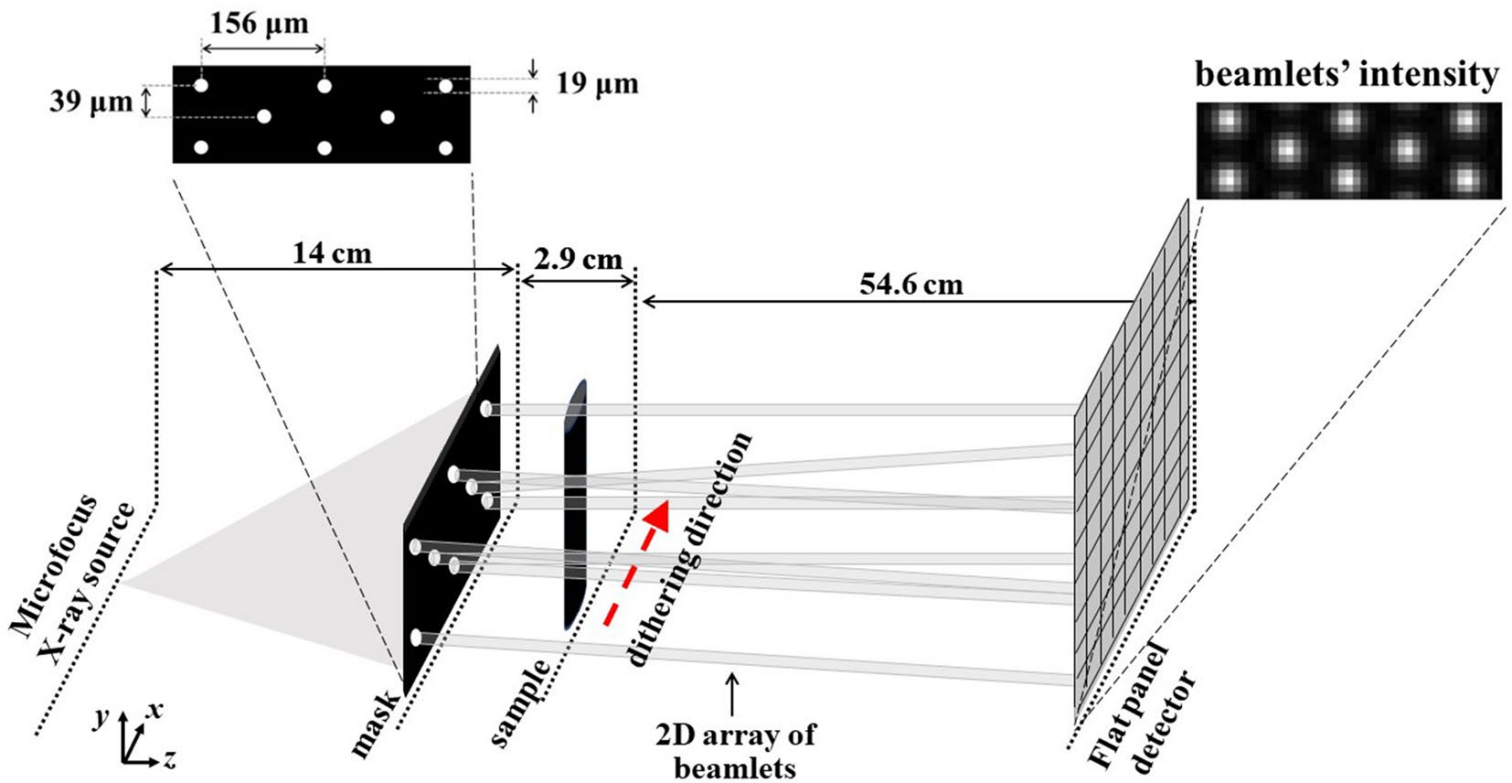
Schematic (not to scale) of the experimental setup. The direction of dithering (sample scanning) is indicated with a red dashed arrow.

A schematic diagram of the 2D beam tracking setup used to acquire the planar images and CT scans is shown in Fig. 1; a description of the setup can be found in the “Methods” section. A mask consisting of a 2D array of circular apertures was used. The apertures have a diameter of *d* = 19 μm and different periods *p*_*h*_ and *p*_*v*_ along the horizontal and vertical direction, respectively; the periods which define the spacing of consecutive beamlets in the respective directions were *p*_*v*_ = 39 μm and *p*_*h*_ = 156 μm. Full details on the mask design are given in the following paragraph.

### Mask design

So long as a sufficient separation between beamlets is achieved, the aperture diameter *d* is the ultimate driver of the system resolution, regardless of the overall system blur *B*_*h,v*_ caused by source and detector. The Gaussian system blur, *B*_*h,v*_, can be obtained by convolving the source distribution *SW*_*h*,*v*_ projected onto the detector with the detector’s Point Spread Function *PSF*_*h,v*_, then backprojecting the resulting function onto to the mask plane,

1 $${B}_{h,v}=\sqrt{{\left(\frac{{SW}_{h,v} \left(m-1\right)}{m}\right)}^{2}+{\left(\frac{{PSF}_{h,v}}{m}\right)}^{2},}$$

where *m* was the magnification factor^26^. An effective separation of the beamlets at the detector is achieved when the cross-sectional sizes of the beamlets, magnified to the detector plane and broadened by the effect of extended source and detector PSF, are smaller than the corresponding magnified periods *p*_*h*_ and *p*_*v*_, i.e.:

2 $$m{p}_{h,v}>\sqrt{{\left(d m\right)}^{2}+{\left({SW}_{h,v}\left(m-1\right)\right)}^{2}+{\left({PSF}_{h,v}\right)}^{2}.}$$

As evident from Eqs. (1) and (2), the mask design is largely determined by the specific experimental setup used. In our case, the focal spot of the x-ray source along the horizontal and vertical axes, *SW*_*h,v*_, was estimated to be approximately 10 μm Full Width at Half Maximum (FWHM). The detector PSF was previously measured to be a Gaussian with 120 μm FWHM along both directions^27^. The magnification factor of the mask, *m*, was 5.11. The system blur (Eq. (1)) is therefore equal to 29 μm in both directions. An aperture diameter of *d* = 19 μm was selected. According to Eq. (2), the FWHM of the beamlets along both directions, demagnified to the mask plane, is 31 μm. Imposing that beamlets overlap with each other at < 10% of their peak value along the vertical direction to allow for their adequate separation, results in a distance between two adjacent apertures along the vertical direction of at least 56 μm. Since as said above the beamlets spread equally in the horizontal and vertical directions, the same separation criterion applies to horizontal spacing between apertures. In our design, we have applied this “minimum 56 μm separation” criterion to a staggered design (offset of half the horizontal period for every other aperture row) where the vertical separation between apertures is smaller than the horizontal one and added a safety margin to (a) de-risk this first proof-of-concept study and (b) be able to use the mask also with other source/detector combinations. We adopted a “safety margin” of approximately 40%, and separated neighbouring beamlets by 78 μm. Achieving isotropic resolution with one-directional dithering also requires the horizontal period to be an integer multiple of the vertical one: all these conditions combined led to the choice of 39 μm for *p*_*v*_ and 4 × *p*_*v*_ = 156 μm for *p*_*h*_. The suitability of such a mask design to fulfil the requirements of the proposed methodology was investigated initially with simulations; a description of the simulation and its results are provided in the Supplementary Materials.

It should also be noted that, for the reasons outlined above, the mask design of this proof-of-concept study does not correspond to complete sample coverage along the vertical direction, as indeed there are gaps between consecutive aperture rows since *p*_*v*_ > *d*. A finer sampling along the vertical direction is possible at the cost of larger aperture spacing in the horizontal direction, and this is also discussed in more detail in the Supplementary Materials.

### Planar images

**Figure 2:**
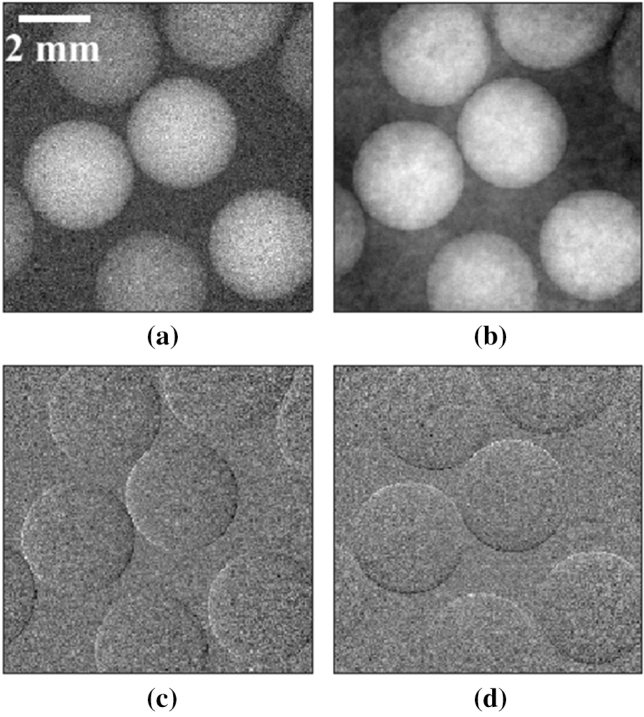
Attenuation (**a**), integrated phase (**b**), refraction along the *x* axis (**c**), and refraction along the *y* axis (**d**), of the spheres sample.

**Figure 3:**
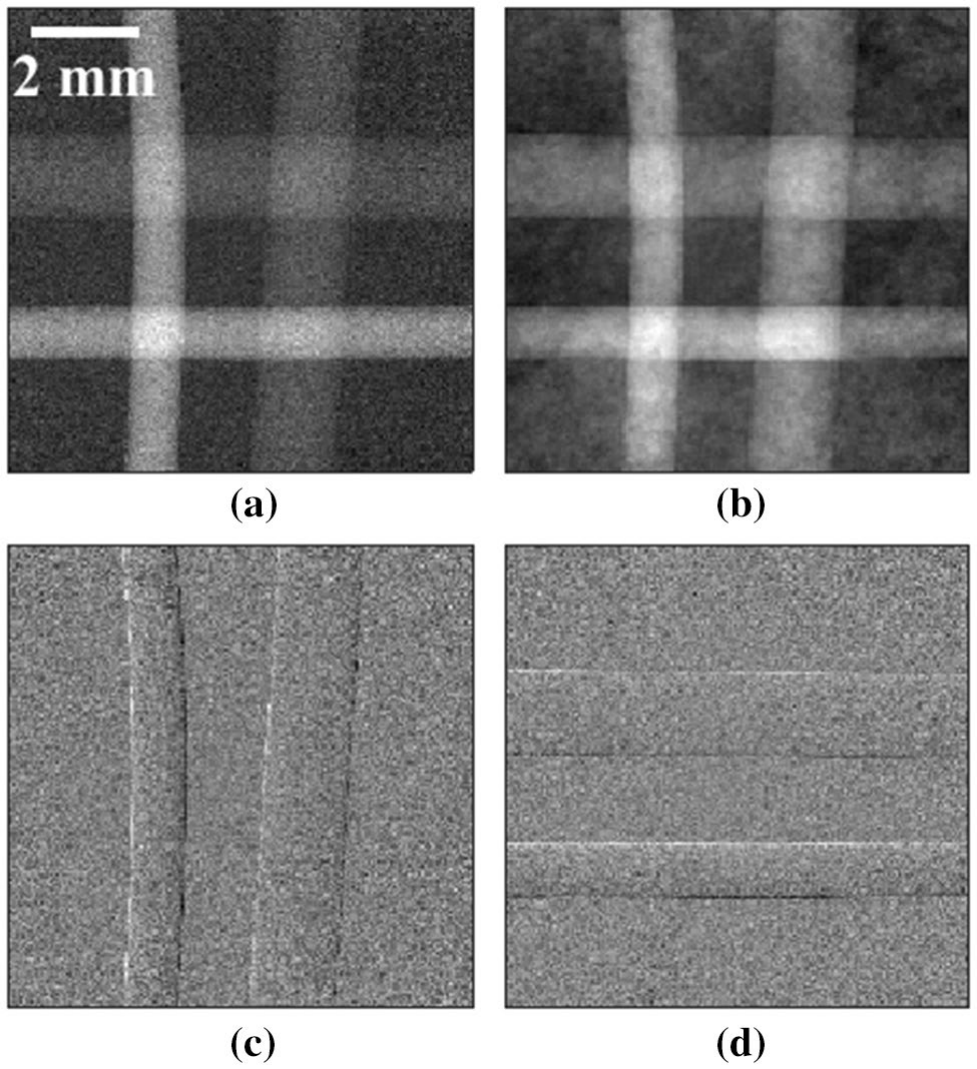
Attenuation (**a**), integrated phase (**b**), refraction along the *x* axis (**c**), and refraction along the *y* axis (**d**), of the crossed wires sample.

Planar images were acquired following the procedure described in the “Methods” section. The retrieved planar images of the spheres and the crossed wires samples are shown in Figs. 2 and 3, respectively. Both these images show attenuation, refraction along the *x* and *y* axis, and integrated phase. A gradient was observed across the retrieved phase images (the background was not constant throughout the images in Figs. 2b and 3b) which was attributed to small errors in the retrieved refraction signals; this is discussed at the end of the section.

**Figure 4:**
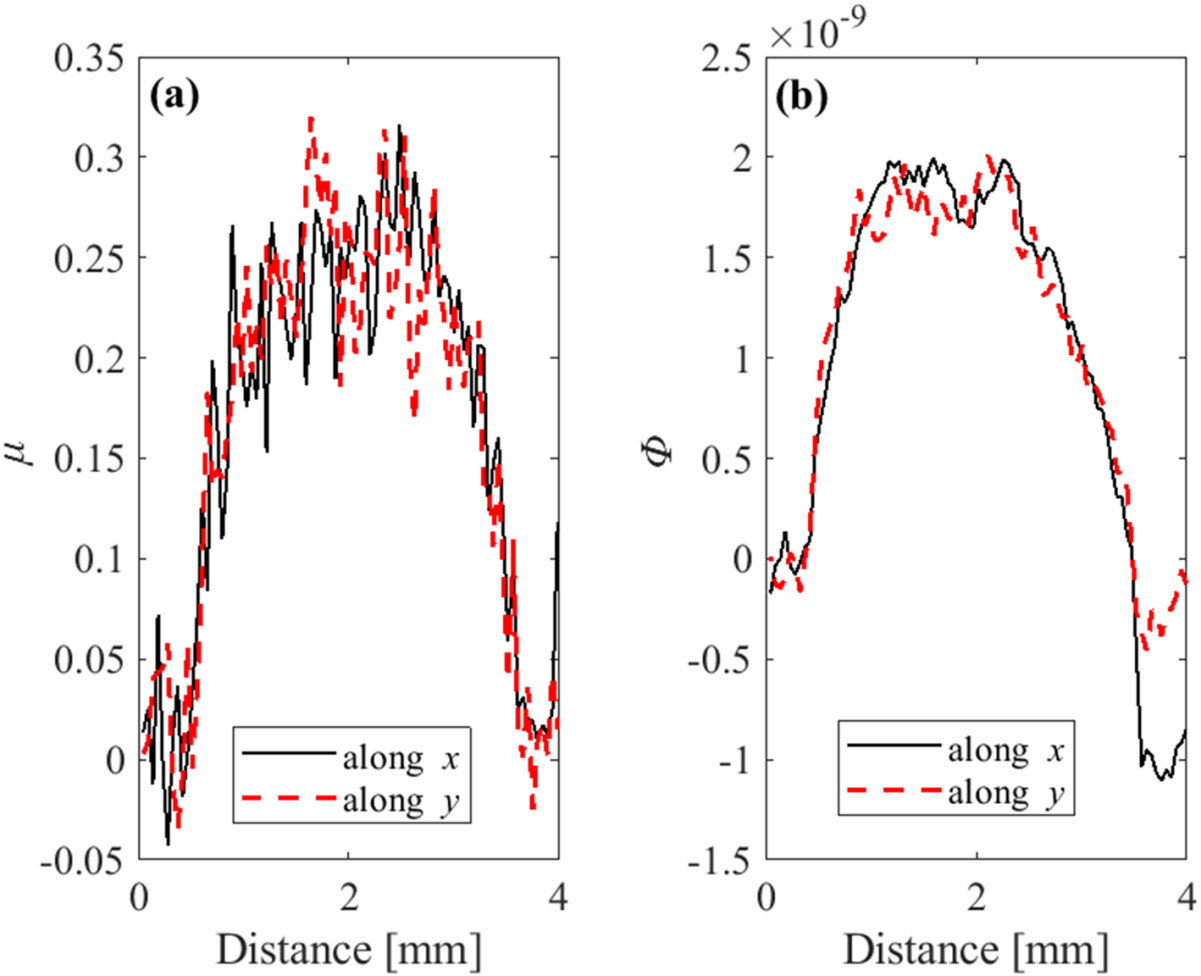
Profiles of attenuation (**a**) and integrated phase (**b**), across the centre of a PMMA sphere (shown in Fig. 2) along the *x* (black solid line) and *y* (red dashed line) direction.

In order to investigate the isotropy of the signals along the horizontal (*x*) and vertical (*y*) direction, the profiles across the centre of a PMMA sphere of the attenuation and phase signals along both directions were plotted and are shown in Fig. 4. It is confirmed that an isotropic spatial resolution across the horizontal and vertical direction is achieved, while dithering along only the horizontal direction was performed.

**Figure 5:**
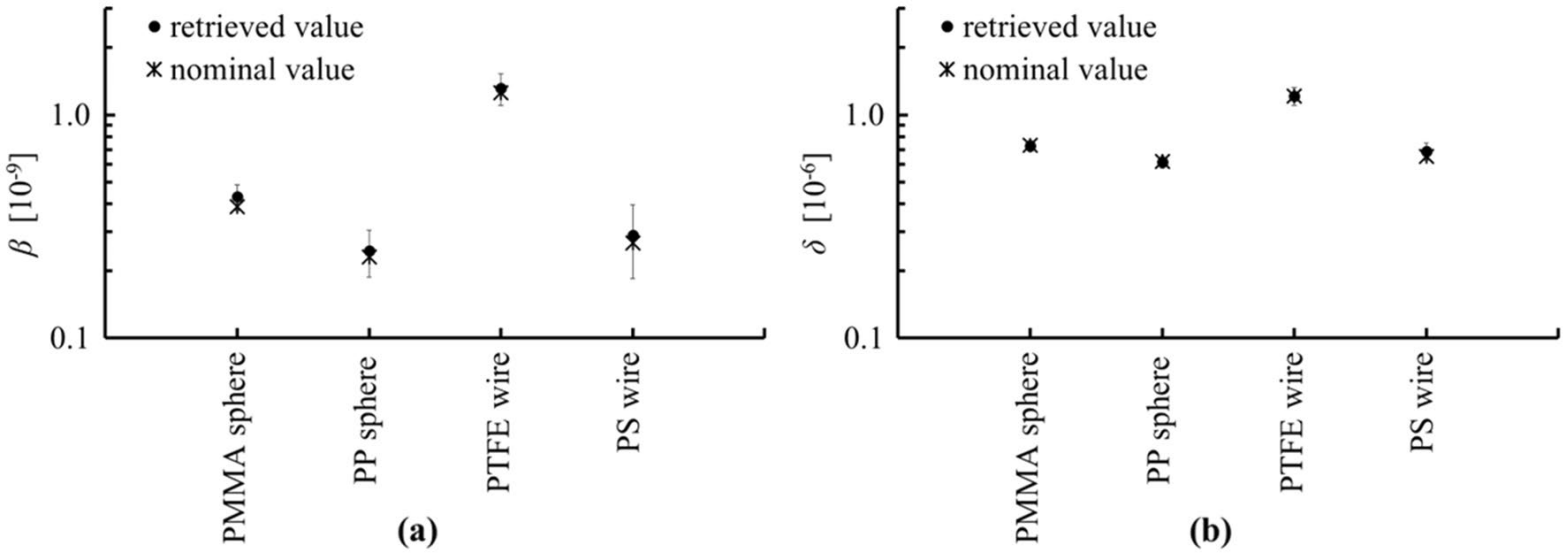
Absorption term, *β*, (**a**) and refraction index decrement, *δ*, (**b**) extracted from the experiment along with the nominal values.

The absorption term *β* and the refractive index decrement *δ*, defined in Eq. (7), of the four materials retrieved as described in the “Methods” section are reported in Fig. 5. The effective energy of the phase measurement, estimated to be approximately 19 keV for the PMMA sphere, PP sphere, and PTFE wire, and 18.5 keV for the PS wire, as described by Munro and Olivo^28^, was used as the mean spectral energy. This same value was used to calculate the retrieved *β* values, leading to recovered values that agree with the nominal ones within uncertainties. In Ref.^28^, Munro and Olivo discuss how the effective energy for absorption may differ from that for phase, and how they both vary with sample thickness. Indeed, the effective energy for the PMMA sphere, PP sphere, and PTFE wire was slightly higher compared to that estimated for the PS wire; this was aligned with the increased absorption of, and therefore beam hardening caused by, the former. It should be noted here that, although a difference of the effective energy between phase and absorption was not observed here, it was believed to be smaller than the uncertainty associated to the retrieved *β* and *δ* values (propagation of the standard deviation of the attenuation and phase values extracted from the images).

### CT scans

**Figure 6:**
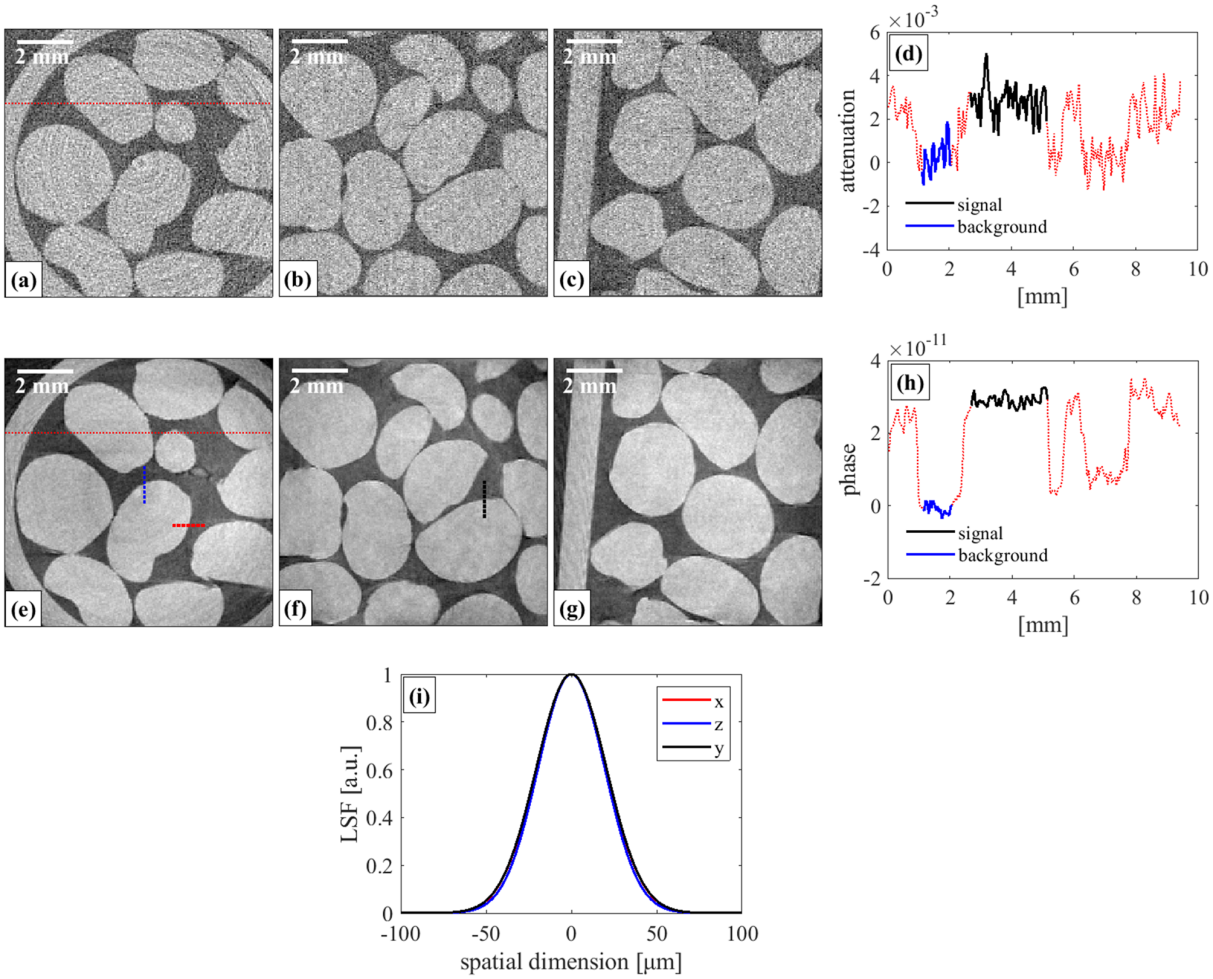
Reconstructed axial (**a**,**e**), sagittal (**b**,**f**), and coronal (**c**,**g**) planes of the granules phantom for attenuation (**a**–**c**) and phase (**e**–**g**), and the corresponding line spread functions (**i**) extracted from the sphere edges indicated with dashed lines of corresponding colours along the *x*, *y* and *z* axes in panels (**e**,**f**). Profiles across the red dotted lines in the axial planes of panels (**a**,**e**) are shown for attenuation (**d**) and phase (**h**).

The reconstructed axial, sagittal, and coronal slices of the granules phantom for both the attenuation and phase channel is shown in Fig. 6; the data acquisition and analysis are described in the “Methods” section.

The first observation from Fig. 6 is the greater contrast and relatively lower noise in the phase images (Fig. 6d–f) compared to the attenuation ones (Fig. 6a–c), The contrast-to-noise ratio (CNR) for attenuation and phase (along the profiles shown in Fig. 6) was calculated to be 3 and 21, respectively. This was attributed to the refractive index decrement *δ* of PS being higher than its absorption term *β* at ~ 19 keV. The second observation is that the spatial resolution appears to be isotropic. Indeed, spatial resolutions (mean ± standard deviation, calculated as per the “Methods” section) of 48 ± 4 μm, 46 ± 5 μm and 48 ± 7 μm, were estimated from the phase volume along the *x*, *z* and *y* axis, respectively, which demonstrates the capability of the proposed mask to achieve isotropic spatial resolution despite one-directional dithering. The line spread functions extracted from the sphere edges along the three axes are shown in Fig. 6 (i). The voxel size, considering the magnification at the sample plane, was 47 μm × 47 μm × 47 μm.

**Figure 7:**
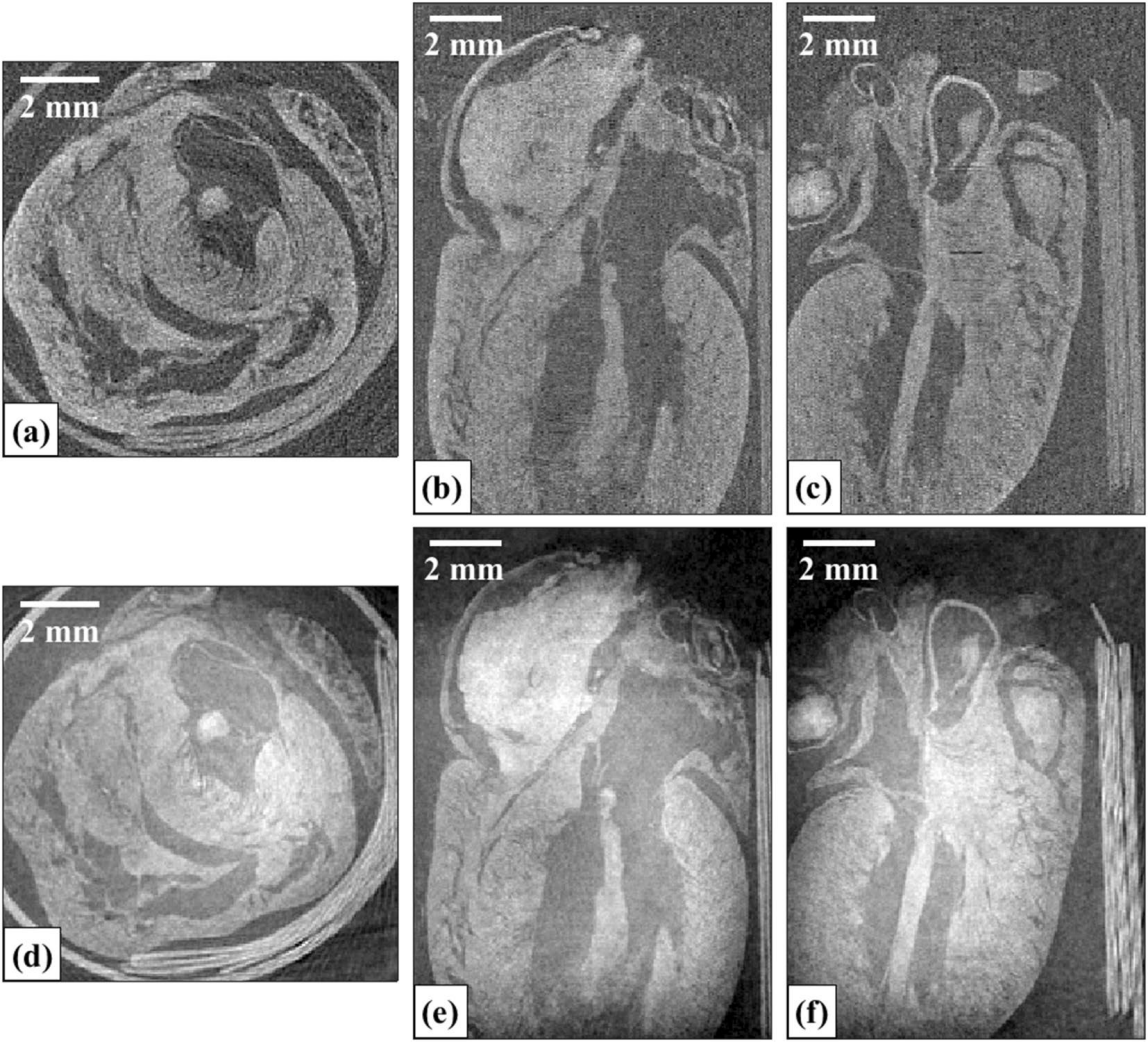
Reconstructed axial (**a**,**d**), sagittal (**b**,**e**), and coronal (**c**,**f**) planes of the rat heart for the attenuation (**a**–**c**) and phase (**d**–**f**) channels.

The compatibility of the proposed methodology for achieving isotropic spatial resolution with unidirectional dithering on a complex biological sample, a rat heart, was also investigated; the data acquisition and analysis is described in the “Methods” section. The reconstructed axial, sagittal, and coronal slices of the rat heart for both the attenuation and phase channels is shown in Fig. 7. As can be seen visually, the spatial resolution appears to be isotropic.

A gradient can be observed across the reconstructed phase slices in Fig. 7, and the phase signal within the heart chambers was higher compared to the background outside the organ. This is due to small errors in the retrieved refraction signals. System instabilities arising from time-varying system components due to e.g., vibrations and/or temperature fluctuations may result in horizontal and vertical shifts of the mask, which can lead to errors in estimating the variation in beamlet position caused by refraction in the sample. Retrieval algorithms based on non-linear curve-fitting solved by the least-square methods, while also accounting for system instabilities, have been shown to remove gradient artefacts in reconstructed phase slices in Edge Illumination x-ray phase contrast CT^29^. Similar retrieval algorithms will be considered in future work for 2D beam tracking x-ray phase contrast imaging and CT.

## Conclusions

A simplified method for achieving two-dimensional phase sensitivity and isotropic spatial resolution is proposed. The proposed setup is a single-grating, beam tracking XPCI/XPC-CT system, which allows the retrieval of attenuation and phase signals from a single frame. Two-dimensional phase sensitivity was achieved by utilizing a mask with a 2D array of circular apertures. An isotropic spatial resolution was achieved by arranging the apertures in a staggered manner, namely by introducing an offset of half the (longer) horizontal period for every other aperture row in a 2D grid with *p*_*v*_ < *p*_*h*_, combined with unidirectional dithering (along the horizontal direction).

The proposed methodology was initially investigated for planar imaging. The retrieved attenuation, refraction along *x* and y and integrated phase images had a square pixel of 47 × 47 μm^2^ size; the attenuation and phase signals were found to be isotropic along the horizontal and vertical direction. Quantitative retrieval of the refractive index decrement *δ* and of the absorption term *β* of the imaged materials was achieved following a previously reported method for polychromatic grating-based x-ray phase contrast imaging systems. This was not repeated for the CT images since the quantitativeness of beam tracking CT was already demonstrated in previous work^17^, and additional literature exist on the subject (e.g.^30^).

This system was then used for XPC-CT. Using a granules phantom, the spatial resolution was estimated to be 48 ± 4 μm, 46 ± 5 μm and 48 ± 7 μm, along the *x*, *z* and *y* axis, respectively, demonstrating that isotropic resolution was achieved. The advantages offered by the phase images compared to the attenuation images, quantified by extracting the CNR values was demonstrated. The suitability of this methodology to image a complex biological sample, a rat heart, was also studied.

In summary, the proposed method offers the potential to achieve single-shot retrieval with two-dimensional phase sensitivity and isotropic spatial resolution with a single optical element and unidirectional dithering. One should note that the approach summarised by Eqs. (1) and (2) determines the minimum separation between beamlets, but it is not prescriptive in terms of their 2D arrangement, therefore leaving room for e.g., a finer sampling along the vertical direction (for example so as to not leave vertical gaps between circular apertures) at the cost of larger circular aperture spacing in the horizontal one (and therefore an increased number of sampling steps). This is discussed in more detail in the Supplementary Materials.

This method offers a simplified acquisition scheme than that required by 2D dithering and is compatible with the smart acquisition scheme for CT, namely cycloidal CT^31^. The latter, which has a more straightforward implementation than its counterpart for 2D dithering, the cycloidal-spiral CT^21^, would decrease even further the acquisition time paving the way for dynamic scans.

## Methods

### Experimental setup

A schematic diagram of the setup is shown in Fig. 1. The x-ray source was a Hamamatsu L12161-07 microfocus source with a W anode, operated in the small focus mode with 40 kV tube voltage and 250 μΑ tube current. The nominal focal spot size at these operating conditions was estimated to be approximately 10 μm FWHM. No beam filtration was used. Following warm-up, the x-ray source was left on for 2 h prior to any acquisition for stabilization purposes. The sample was placed at 16.9 cm from the source on a sample stage, consisting of Physik Instrumente (PI) Piezo Motors, for rotation (model Q-632.930) and linear translation (model Q-521.240, three in total, one for each direction, plus a Newport linear stage (M-ILS150BPP) to perform the (horizontal) dithering. A 30 × 30 mm^2^ mask was placed 2.9 cm upstream of the sample. It consisted of 19 μm circular apertures with a period of 156 μm along the horizontal and 39 μm along the vertical direction, with an offset of half the horizontal period (i.e., 78 μm) every other line. It had a 200 ± 20 μm thick Au layer on a 1 mm thick graphite substrate, and was fabricated by Microworks GmbH (Karlsruhe, Germany) to the authors’ design (described in more detail below). The detector was a Hamamatsu CMOS-based flat panel sensor (model C9732DK) with 2368 (h) × 2340 (v) 50 × 50 μm^2^ pixels. The angular filtration caused by the use of a relatively thick flat mask with a cone beam led to an effective field of view of 15.9 (h) × 9.4 (v) mm^2^. A mask-to-detector distance of 57.5 cm was used, resulting in a magnification of the mask equal to 5.11; the horizontal period of the beamlets at the detector was 16 pixels. The sample-to-detector distance was thus 54.6 cm, with a sample magnification of 4.23.

### Data acquisition

A total of two planar images followed by two CT scans were acquired. The two samples for the planar images were (1) 4 × 3.5 mm diameter polystyrene (PS), 4 × 3.18 mm diameter polypropylene (PP) and 4 × 3.18 mm diameter polymethyl methacrylate (PMMA) spheres enclosed in a membrane film box (referred to as spheres sample hereafter), and (2) 2 × 1 mm diameter polytetrafluoroethylene (PTFE) wire and 2 × 1.6 mm diameter PS rods, arranged in a crossed fashion and enclosed in a membrane film box (referred to as wires sample hereafter). For each sample, 30 dark and 30 flat images were acquired, followed by the sample images. The sample was translated along the horizontal direction in 4 dithering steps, covering one horizontal mask period (156 μm). Considering the 1.21 sample-to-mask magnification, the sample covered 188 μm in 4 × 47 μm dithering steps.

The two samples for the CT scans were: (1) a series of approximately 3.5 mm diameter PS granules inserted in a 10 mm diameter plastic straw (referred to as granules phantom hereafter), and (2) a freeze-dried rat heart (kept at room temperature during the scan). For each sample, 30 dark and 30 flat images were acquired before and after the acquisition of the sample images. The dithered CT dataset consisted of 1008 projections taken by rotating the sample in steps of 0.18 degrees over 180 degrees plus the cone angle, here equal to 1.4°, in a “step-and-shoot” fashion. At each angle, the sample was scanned horizontally in 4 × 47 μm steps, and a 1.2 s exposure frame was acquired at each step. This led to a total of 4032 frames for each CT scan, with a total duration (including overheads arising from the step-and-shoot nature) of ~ 390 min.

### Data analysis

Each frame was initially dark corrected. Attenuation and refraction along *x* and *y* signals were then retrieved from each frame, by tracking each beamlet’s profile and quantifying the changes induced by the sample. More specifically, the beamlet’s intensity with and without the sample, *I* and *I*_*0*_ respectively, were quantified; the reduction of the beamlet’s intensity was then related to the x-ray attenuation, through:

3 $$-\mathrm{ln}\frac{I}{{I}_{0}}=\mu ,$$

where

4 $$\mu =2\frac{2\uppi }{\lambda }\int \beta (x,y,z)\mathrm{d}z.$$

In Eq. (4), *λ* is the x-ray wavelength, *β* the absorption term of the complex refractive index, and z is the direction of x-ray beam propagation. The horizontal and vertical displacements of the beamlet, Δ*Sx* and Δ*Sy*, respectively, were tracked using subpixel image registration based on cross correlation^32^. These displacements were due to refraction and were related to the refraction angle along the horizontal, *θ*_*Rx*_, and vertical, *θ*_*Ry*_, directions by:

5a $${\theta }_{Rx}={\mathrm{tan}}^{-1}\left(\frac{\Delta {S}_{x}}{{z}_{sd}}\right),$$

and

5b $${\theta }_{Ry}={\mathrm{tan}}^{-1}\left(\frac{\Delta {S}_{y}}{{z}_{sd}}\right).$$

Following attenuation and (*x* and *y*) refraction retrieval from each frame, the four frames for each channel corresponding to the four dithering steps were combined into a single image, with four times as many pixels in the *x* direction as the original retrieved images. The phase shift *Φ* induced by the sample was retrieved using the refraction angles *θ*_*Rx*_ and *θ*_*Rx*_ and the Fourier space method described in^33^. *Φ* is related to the refractive index decrement *δ* through:

6 $$-\Phi =k\int \delta \left(x,y,z\right)\mathrm{d}z,$$

where *k* is the wavenumber. For the quantitative analysis, the unit decrement *δ* and the absorption term *β* of the complex refractive index:

7 $$n\left(E\right)=1-\delta \left(E\right)+i\beta \left(E\right),$$

were retrieved for the four materials in the acquired planar images of the spheres and wires samples. This was obtained from the attenuation images by rearranging Eq. (4) as:

8 $$\beta = \frac{\mu }{2 }\frac{\lambda }{2\uppi }\frac{1}{T},$$

and from the phase images by rearranging Eq. (6) as:

9 $$\delta =\frac{\Phi }{T},$$

where *T* is the thickness of the sample along the direction of the x-ray beam propagation. The mean and standard deviation (SD) of the attenuation and phase values were calculated from regions of interest selected within each material (PP and PMMA spheres and PTFE and PS wires) in the corresponding images. *β* and *δ* were then calculated using Eqs. (8) and (9), with their SD calculated through standard propagation of the SD values extracted from the images. The effective energy of the phase measurements, estimated from the comparison of the *δ* values retrieved for each material (using Eq. (9)) to their energy-dependent nominal values (extracted using xraylib^34^), was used as representative of the polychromatic spectrum, and subsequently in Eq. (8) to retrieve the *β* values.

CT reconstruction of both the attenuation and phase images was performed with a GPU implementation of the Feldkamp-David-Kress algorithm^35^ for cone-beam reconstruction using the ASTRA toolbox^36,37^. The reconstructed planes had a pixel area equal to the horizontal dithering step at the sample plane × the vertical period of the beamlets at the sample plane, i.e. 47 × 47 μm^2^.

The reconstructed axial, sagittal, and coronal planes of the granules phantom were used to estimate the spatial resolution of the system. Spatial resolution estimates were obtained by fitting error functions to the edges of granules along the *x*, *y*, and *z* directions, computing their derivatives to obtain line spread functions (LSF), and extracting their resulting full width at half maxima (FWHM). Five consecutive edges were fitted for each direction and the mean and SD values calculated. It should be noted that, even though the edges of the granules were not strictly sharp, profiles were extracted from CT slices at their centre, and these can be considered to have negligible curvature when considering their overall size (approximately 3.5 mm diameter) compared to the thickness (47 μm) of the reconstructed CT slices.

The reconstructed attenuation and phase axial planes of the granules phantom was also used to quantify the CNR for each contrast channel. Attenuation and phase profiles were extracted from a selected region within the rat heart; the signal and the background regions were identified. CNR was then calculated as follows

10 $$CNR= \frac{{I}_{signal}-{I}_{background}}{{\sigma }_{background}}$$

where *I* denotes the mean and *σ* the SD of each region.

## Supplementary Information

Supplementary Information.

## Data Availability

The data that support the findings of this study are available from the corresponding author upon reasonable request.
