# Supplementary material for: A laboratory-based beam tracking x-ray imaging method achieving two-dimensional phase sensitivity and isotropic resolution with unidirectional undersampling
Source: Sci Rep. 2023 May 29;13:8707. doi: 10.1038/s41598-023-35901-2 (PMC10227046; doi:10.1038/s41598-023-35901-2)
Supplement: Supplementary file 1 — Supplementary Information. [file 41598_2023_35901_MOESM1_ESM.docx]

**Supplementary materials**

**Simulation studies used for mask design**

The experiment was initially modelled using a wave optics simulation. The simulation code available to our group, described in detail in Ref. [1], was expanded to simulate two-dimensional systems. In brief, the original wave optics model simulates the free-space propagation of x-rays between two parallel planes using the Fresnel-Kirchoff theory of diffraction in the Fresnel approximation; the complex amplitude of the electric field at the latter plane is calculated as the convolution between the complex amplitude of the electric field at the initial plane and the Fresnel propagator [1]. The mask and the object are modelled as complex transmission functions. The detected signal is calculated by taking into account the x-ray source distribution and the detector PSF through additional convolutions. In this original version, each function (i.e. complex transmission functions of mask and object, x-ray source distribution, detector PSF, and Fresnel propagator) is sampled along the *x* direction (horizontal) only, at a 1/Δ*x* sampling rate and along a length L*x*. Hence, a 1D imaging system (i.e. a single row of pixels, with no dependency along the *y* direction) is simulated.

Here, this model was extended from 1D to 2D. Fresnel propagator, x-ray source distribution, detector PSF, mask, and object were all defined using 2D functions, with dependency along both the *x* and *y* directions. Each function was then sampled along the *x* and *y* direction at 1/Δ*x* and 1/Δ*y* sampling rates, over an area *L_x_* × *L_y_*. Two-dimensional Fourier transforms were used and the integration of the calculated amplitude at the detector plane over the entire pixel area was performed.

The experimental setup described in the main article (Fig. 1) was simulated, while introducing a 3.2 mm diameter PMMA sphere downstream of the mask. A sampling step of Δ*x* = Δ*y* = 500 nm was used to sample all functions along both directions. A monoenergetic x-ray source of 19 keV and a detector size of 449 (*h*) × 377 (*v*) pixels were simulated. A total of four images of the sample were acquired, one for each dithering step; the sample was stepped along the horizontal direction with a step size of ¼ of the horizontal mask period for each dithering step. The retrieval of the three signals was then performed as described in the Methods section of the main article.

The flat field was initially simulated to confirm that sufficient beamlet separation could be achieved. The results are presented in in Supplementary Fig. 1 (a)-(c), alongside their experimental correspondents (panels (d)-(f)). Alongside the good agreement between simulation and experiment, the figure also shows how the selected mask design allowed the beamlets to be as close as possible in the vertical direction with minimal overlap.


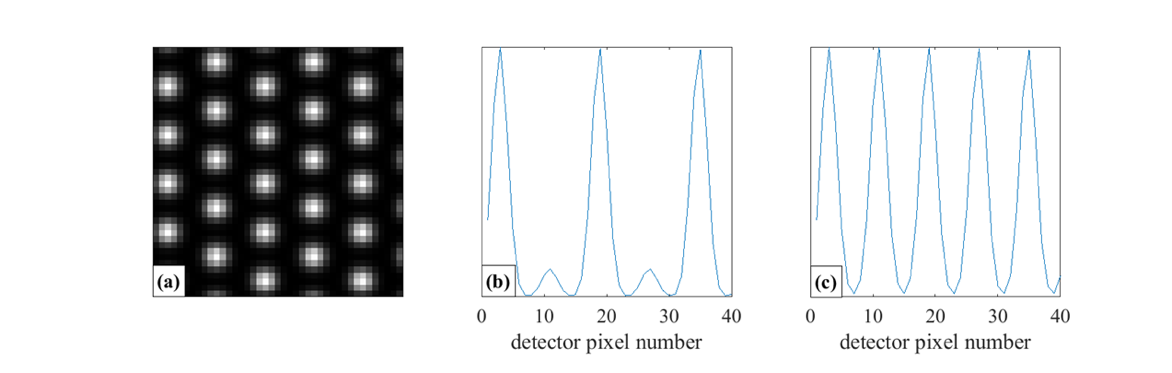


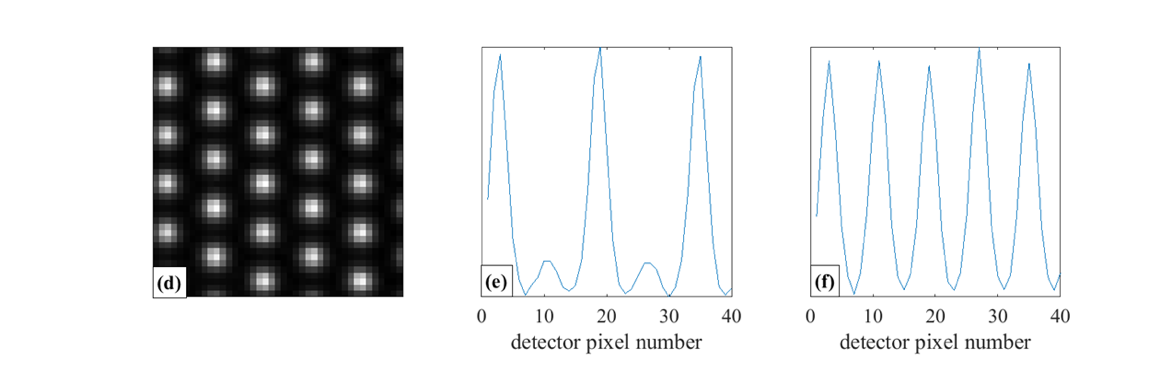


**Supplementary Fig. 1.** Simulated (a-c) and experimentally measured (d-e) beamlets’ intensity at the detector; sample-less images ((a) and (d)), intensity along the horizontal axis *x* ((b) and (e)), and intensity along the vertical axis *y* ((c) and (f)) are shown.

The retrieved and up-sampled (combining the four dithering steps) images of the PMMA sphere can be seen in Supplementary Fig. 2. Attenuation, integrated phase and refraction along the *x* and *y* axis are shown in panels (a)-(d), respectively.


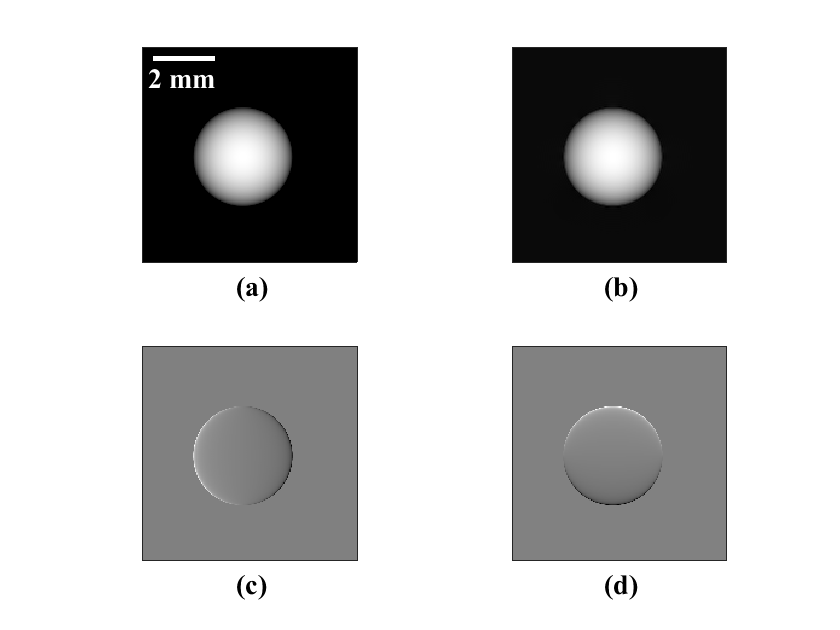


**Supplementary Fig. 2.** Simulated attenuation (a), integrated phase (b), refraction along the *x* axis (c), and refraction along the *y* axis (d), of the PMMA sphere.

In order to verify the isotropy of the retrieved signals along the two directions, the profiles of the attenuation, phase, and refraction signals across the centre of the sphere along the *x* (horizontal) and *y* (vertical) direction were plotted; these are shown in Supplementary Fig. 3, which confirm that good isotropy is achieved. This is in accordance to the findings from the experimental image of a PMMA sphere, the profiles of which are shown in Fig. 4 of the main article.


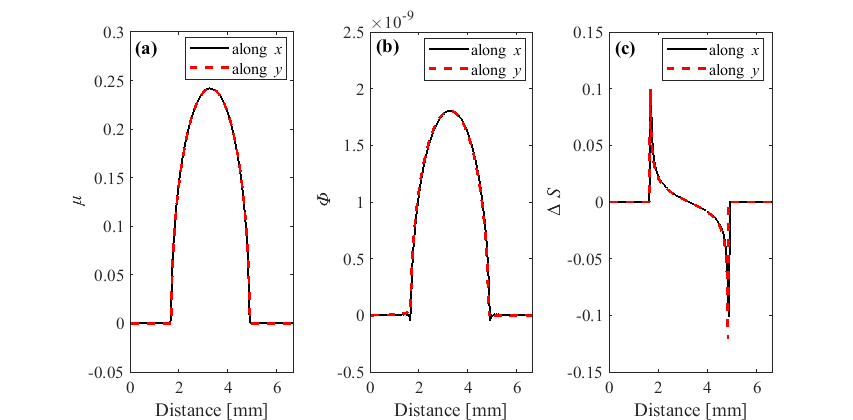


**Supplementary Fig. 3.** Simulated profiles of attenuation (a), integrated phase (b), and refraction (c), across the centre of the PMMA sphere along the *x* (black solid line) and *y* (red dashed line) direction.

**Differences from sampling schemes based on parallel slits and circular apertures arranged in square grids**

The mask was designed such that 1) two-dimensional phase sensitivity is achieved and 2) undersampling is much more significant along the horizontal direction. The first requirement is fulfilled by using a mask with a 2D array of circular apertures. The second requirement is fulfilled by having different periods along the two directions and replacing the vertical dithering with the mask’s shorter period (*p_v_*). As a result, a unidirectional (along the horizontal, i.e., *x*, direction) dithering is sufficient for sampling with the desired pitch.

To allow their effective tracking of each beamlet, their overlap (along any direction) should be minimised. In order to minimize the vertical period while keeping the beamlets separated for effective tracking, a longer horizontal period was adopted, and adjacent rows of circular apertures were offset with respect to each other, by distributing the circular apertures in a staggered manner. The horizontal period was a multiple of the vertical period such that, when a horizontal dithering step equal to the vertical period is applied, the retrieved images have square pixels, leading to isotropic resolution as demonstrated in the main article.

The projection sampling grid adopted here is depicted in Supplementary Fig. 4(c). For comparison purposes, previously adopted sampling grids for beam tracking XPCI/XPC-CT are also shown in Supplementary Fig. 4: these were one-dimensional phase sensitivity with one-dimensional undersampling (mask with slits) [2] and two-dimensional phase sensitivity with two-dimensional undersampling (mask with circular apertures in a square grid) [3].


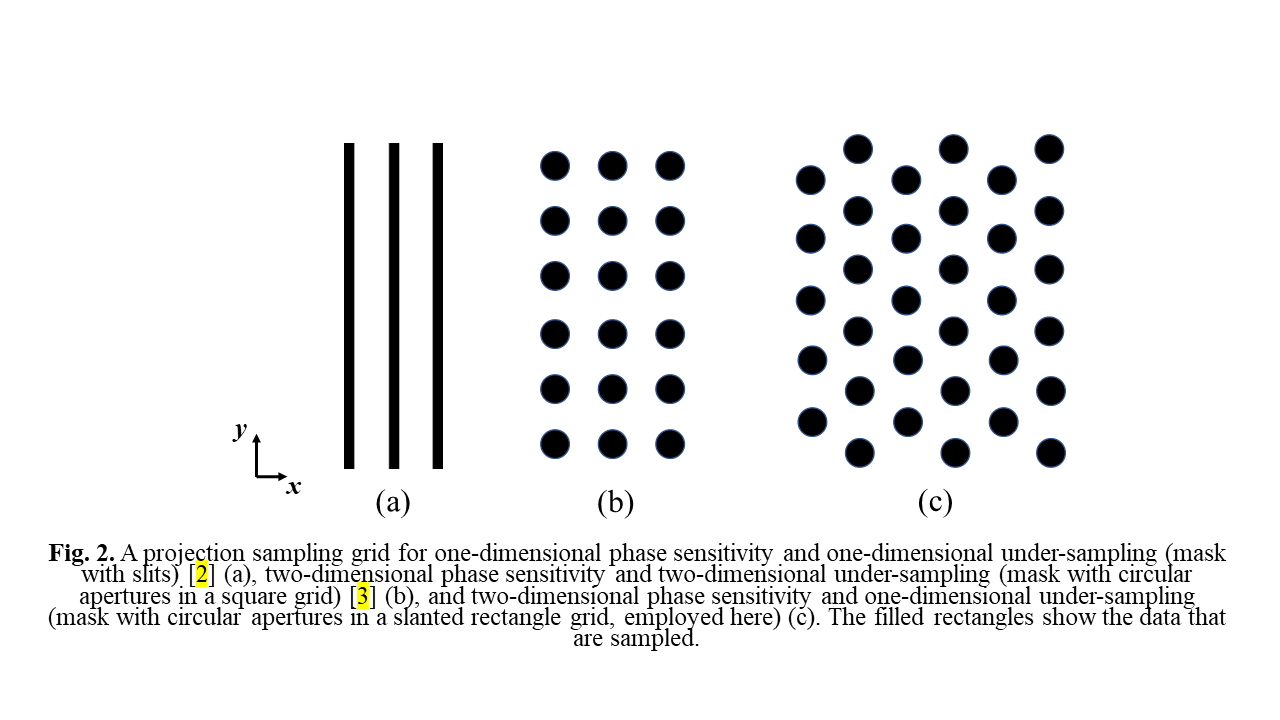


**Supplementary Fig. 4.** A projection sampling grid for one-dimensional phase sensitivity and one-dimensional undersampling (mask with slits) [2] (a), two-dimensional phase sensitivity and two-dimensional undersampling (mask with circular apertures in a square grid) [3] (b), and two-dimensional phase sensitivity and one-dimensional undersampling (mask with circular apertures in a staggered rectangle grid, employed here) (c). The filled rectangles/circles show the data that are sampled.

As discussed in the main article, for this first proof-of-concept study a vertical gap was left between adjacent rows of apertures. However, this is not necessary. Equations (1) and (2) in the main paper allow calculating the minimum distance *d_m_* between two apertures that allows a sufficient separation between adjacent beams to enable effective attenuation, refraction and scatter retrieval. Therefore circular apertures can be arranged as shown in Supplementary Fig. 5(b).


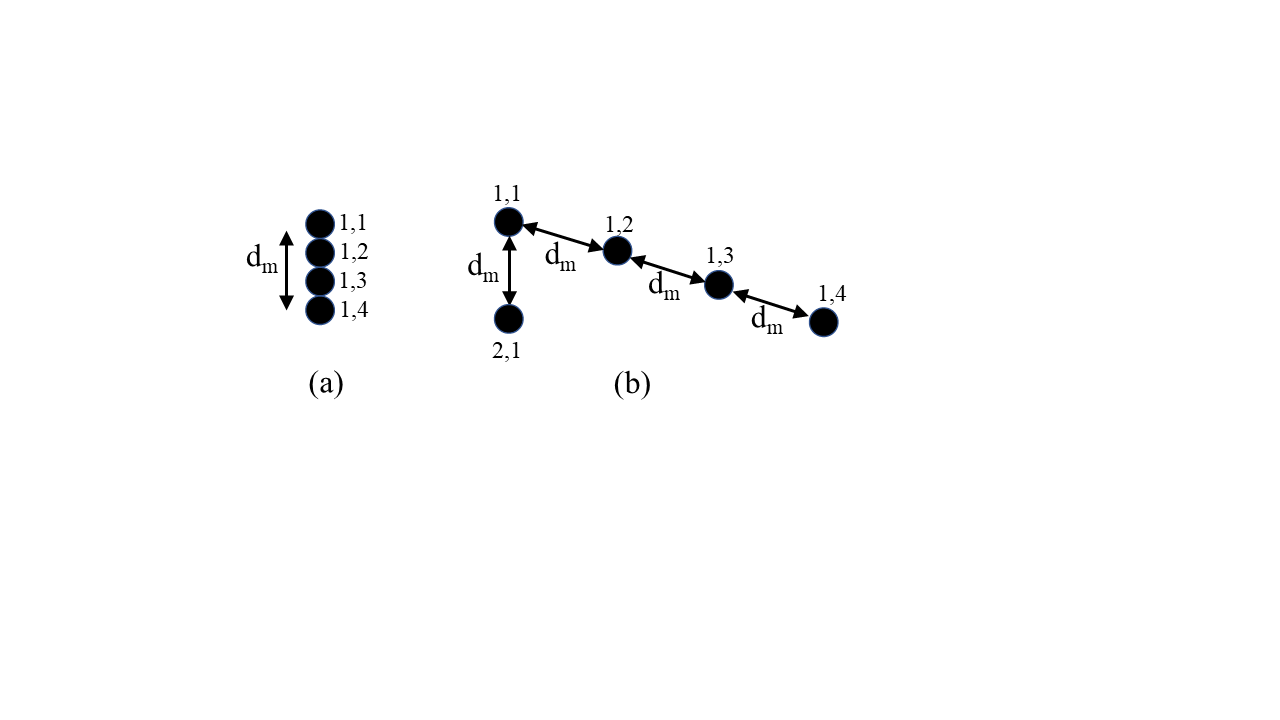


**Supplementary Fig. 5**: Example of circular aperture arrangement allowing full sample coverage with unidirectional scanning

Supplementary Fig. 5(a) shows a “dense” arrangement of apertures that, if scanned horizontally across a sample, allows its full coverage. This is not feasible in practice because the beamlets would overlap at the detector making attenuation, refraction and scatter retrieval impossible. To effectively allow such a retrieval, individual apertures must be shifted horizontally until their diagonal distance is equal to *d_m_*, as shown in Supplementary Fig. 5(b). This does not change their vertical distance, hence full sample coverage is still achieved. The process continues until the vertical distance between “displaced” rows of apertures is also equal to *d_m_*; at that point, aperture 2,1 can be placed directly under 1,1 as shown in Supplementary figure 5(b) (note that in this simplified explanation aperture 1,4 would effectively correspond to 2,2, which was ignored for ease of explanation). This recipe allows calculating the aperture arrangement on the basis on simple geometric considerations; it should be noted that alternative arrangements are also possible, with the one in Supplementary figure 5(b) being the simplest and therefore the best suited for explanation purposes. Utilizing a standard mask, e.g., the one enabling the sampling grid shown in Supplementary Fig. 4(b), and tilting it around the optical axis (*z* direction) would result in the apertures being arranged in a staggered grid, however, the approach followed here (Supplementary Fig. 4(c)): 1) enables an optimized aperture separation along the horizontal and vertical axis and 2) ensures a square or rectangular FoV.

**Supplementary References**

[1] Vittoria, F. A. *et al*. Strategies for efficient and fast wave optics simulation of coded-aperture and other x-ray phase-contrast imaging methods. *Appl. Opt.* **52**, 6940 (2013).

[2] Vittoria, F. A., *et al.* Beam tracking approach for single–shot retrieval of absorption, refraction, and dark–field signals with laboratory x–ray sources. *Appl. Phys. Lett.* **106**, 224102 (2015).

[3] Navarrete-León, C. *et al*. arXiv:2212.07963 (2022).
